# Obesity: Rubensian beauty turned into major health problem

**DOI:** 10.3325/cmj.2017.58.89

**Published:** 2017-04

**Authors:** Sandor G. Vari

**Affiliations:** 1 International Research and Innovation in Medicine Program, Cedars-Sinai Medical Center, Los Angeles, CA, USA & Association for Regional Cooperation in the Fields of Health, Science and Technology (RECOOP HST Association), Hungary *vari@cshs.org*

## Beauty and the belly

The Dutch painter Sir Peter Paul Rubens (June 28, 1577 – May 30, 1640), the most famous artist of the Flemish Baroque art school, studied anatomy and medicine to understand the human body and devoted his life to sharing views common among his contemporaries. Rubens believed that physical weakness could lead to spiritual weakness. He painted nudes and highlighted their physical beauty, goodness, temptation, desire, and fertility. His full-figured women were later called “Rubenesque” or “Rubensian” (1). His most famous “Rubensian” paintings are Venus, Cupid, Bacchus, and Ceres (painted in 1612), Amor and Venus (1614), Venus at the Mirror (1615), and The Three Graces (1635). Today Rubens is more widely known as the painter of “big” women than for his religious and mythological paintings, portraits, self-portraits, and landscapes (2).

## “Rubensian” body type is apple shaped

Both developed and developing countries are suffering from obesity or a “Rubensian” epidemic and we must face its health and economic consequences at local, national, and global levels (3). In 2017, the proportion of overweight or obese individuals has increased approximately 10% (from 66.7% to 76.8%) for men, and 9% (from 54.8% to 63.4%) for women compared with data for 1991–93 and 2011–13 in the UK (4). An excess of visceral fat is known as central obesity; the abdomen protrudes excessively as the “pot belly” or “beer belly”. This body type is also known as “apple shaped” as opposed to “pear shaped” in which fat is deposited on the hips and buttocks. Scientists have come to recognize that body fat rather than body weight is the key to evaluating obesity (5).

In healthy, non-overweight humans, white adipose tissue (WAT) accounts for as much as 20% of the body weight in men and 25% in women. The brown adipose tissue (BAT) contains multiple lipid droplets, high density vasculature, and mitochondrial cytochromes (6). WAT and BAT have antagonistic functions. WAT stores surplus energy as triglycerides, and an excessive volume of WAT is associated with increased risk of obesity-related disorders. BAT is specialized for releasing energy through the production of heat during adaptive thermogenesis and is associated with a decreased risk of obesity-related disorders. WAT is the main component of the abdominal obesity type, colloquially known as belly fat. Intra-abdominal obesity, or visceral or *intra-abdominal fat*, is fat tissue stored around abdominal and thoracic organs, as opposed to subcutaneous fat (located underneath the skin) or intramuscular fat (interposed in skeletal muscle) (5,7). BAT is mainly stored in mediastinal, pericardial, para-aortic, interscapular, and supraclavicular areas and around the kidneys and pancreas. BAT decreases with the increase in body weight and advanced age (8,9). Adipose tissue can expand many-fold during adulthood and is evaluated by the waist-hip ratio (WHR) and body mass index (BMI).

## Adipose tissue is acting as an organ

Accumulation of adipose tissue in individuals with obesity is associated with a state of persistent low-grade inflammation that seems to play a pivotal role in the pathogenesis of obesity-linked insulin resistance, diabetes, and cardiovascular and neurodegenerative diseases (10). In this condition, adipose tissue is no longer considered only as a fat storage, but as an endocrine organ responsible for the synthesis and secretion of several hormones. Leptin and renin-angiotensin system control nutritional intake and insulin sensitivity. Tumor necrosis factor α (TNF-α), interleukin-6 (IL-6), resistin, visfatin, and adiponectin are mediators of the inflammatory process responsible for persistent low-grade inflammation (11).

## Death sentence written on the belly

Obesity may not be the Black Death (the plague disease, caused by *Yersinia pestis*), nevertheless, it is a public health crisis. The Black Death was one of the most devastating pandemics in Eurasia; in the period between 1346 and 1353, an estimated 75 to 200 million people died (12,13).

Most of the world’s population live in countries where being overweight or obese kills more people than other diseases. Since 1980, obesity has doubled worldwide; in 2014, over 600 million people were obese, ie, 13% of the world population, and more than 1.9 billion adults were overweight, of whom 39% were aged 18 years or older (14).

The number of people with diabetes mellitus is increasing as the incidence of obesity is increasing. The World Health Organization (WHO) reported that the number of people with diabetes mellitus increased 4-fold from 1980 to 2014. In 2012, 1.5 million deaths were caused directly by diabetes and 2.2 million deaths were attributable to high blood glucose. Ten years from now, the 7th leading cause of death will be diabetes. The WHO estimated 108 million people lived with diabetes in 1980 and reported 422 million diabetic people in 2014. Diabetes has become a global issue and, although the number of “Rubensians” is rapidly increasing in high-income countries, we can also observe the same trend in middle- and low-income countries (15). One of the most devastating consequences of diabetes is the lower extremity amputation (LEA). The development of atherosclerosis can lead to vessel blockade and limb amputation becomes necessary because arterial reconstruction would not lead to a better result (16,17).

The major complications of diabetes are heart attack, stroke, kidney failure, blindness, and neurodegenerative diseases. In 2012, an estimated 17.5 million people died from cardiovascular diseases (CVD), 7.4 million from coronary heart disease, and 6.7 million from stroke. Globally, more people die annually from CVD than from any other cause; CVD is responsible for 31% of global deaths. Three quarters of CVD deaths occurred in low- and middle-income countries (18).

The coming epidemic is neurodegenerative diseases. By 2025, the percentage of people in the EU population aged over 65 years is predicted to increase from 15.4% to 22.4%, which is likely to correlate with a rise in Alzheimer disease (AD), accounting for one-half to three-quarters of all dementia cases (19). Midlife obesity can lead to the development of AD, the silent killer of nerve cells. AD is also called “type 3 diabetes” because obese people with metabolic syndrome (also known as insulin resistance syndrome) are at higher risk for developing the disease (20). AD starts damaging the brain quietly more than a decade before the first symptoms appear. Lipid rafts are one of the key players in the pathogenesis of several neurodegenerative diseases, including AD. Lipid rafts represent a platform for protein-lipid and protein-protein interactions and have roles in cellular signaling. Lipid rafts are responsible for generation of the amyloid β peptide. As “Rubensian” individuals get older, their brain tissue has fewer nerve cells and synapses in comparison with a healthy brain, and abnormal clusters of protein fragments called plaques build up between nerve cells (21).

## Reducing the “Rubensian” beauty belly and diabetes

One of the most commonly used oral antidiabetic medicines for the treatment of insulin-resistant diabetes is metformin. Metformin stimulates AMP-activated protein kinase (AMPK) that inhibits lipid synthesis through phosphorylation and inactivation of key lipogenic enzymes (22). It could reduce visceral fat probably through a mechanism that shifts fat oxidation in WAT as surplus energy and up-regulates the adaptive thermogenesis in BAT. Therefore, metformin could be a good candidate for the treatment of a calorie surplus or age-related obesity (23).

Liraglutide, another widely used antidiabetic drug in type 2 diabetes, is an incretin mimetic and helps the pancreas to release the right amount of insulin after consumption of carbohydrates. Liraglutide is also successfully used in non-diabetic obesity for weight loss (24). Its mechanism in weight loss not completely understood but it is known that liraglutide inhibits the glucagon release, decreases food intake through the early sensation of satiety, delays gastric emptying, and reduces WAT accumulation through increasing lipolysis of adipocytes (25).

The “Rubensian beauty” is not merely an individual problem. It has become epidemic! New approaches are required to investigate the complex origin of obesity and related diseases, as obesity rates continue to rise in both the developed and the developing world.
